# Advances in retina imaging as potential biomarkers for early diagnosis of Alzheimer’s disease

**DOI:** 10.1186/s40035-021-00230-9

**Published:** 2021-02-01

**Authors:** Ying Zhang, Yanjiang Wang, Ce Shi, Meixiao Shen, Fan Lu

**Affiliations:** 1 School of Ophthalmology and Optometry, Wenzhou Medical College, Wenzhou, 325027 China; 2 Department of Neurology, Daping Hospital, Third Military Medical University, Chongqing, 400042 China

**Keywords:** Alzheimer’s disease, Mild cognitive impairment, Optical coherence tomography, Optical coherence tomography angiography, Retina, Biomarkers, *In vivo* imaging

## Abstract

As the most common form of dementia, Alzheimer’s disease (AD) is characterized by progressive cognitive impairments and constitutes a major social burden. Currently, the invasiveness and high costs of tests have limited the early detection and intervention of the disease. As a unique window of the brain, retinal changes can reflect the pathology of the brain. In this review, we summarize current understanding of retinal structures in AD, mild cognitive impairment (MCI) and preclinical AD, focusing on neurodegeneration and microvascular changes measured using optical coherence tomography (OCT) and optical coherence tomography angiography (OCTA) technologies. The literature suggests that the impairment of retinal microvascular network and neural microstructure exists in AD, MCI and even preclinical AD. These findings provide valuable insights into a better understanding of disease pathogenesis and demonstrate that retinal changes are potential biomarkers for early diagnosis of AD and monitoring of disease progression.

## Background

Alzheimer’s disease (AD) is a leading cause of dementia and the most common chronic neurodegenerative disease leading to cognitive impairment in the elderly. AD is pathologically characterized by abnormal extracellular senile plaques consisting of amyloid-beta (Aβ) peptide and intracellular neurofibrillary tangles composed of hyperphosphorylated tau protein. Other disease-specific signs include cerebral amyloid angiopathy resulting from amyloid deposition on microvascular walls, neuron and synaptic loss, inflammation, and gliosis [1–4]. It has been estimated that by 2050 up to 131 million people would suffer from AD [5]. Despite the advances in understanding the pathogenesis and clinical practice over the past decades, current clinical treatments are limited as most patients have advanced AD with irreversible neuronal damage. It is generally believed that early intervention can slow the progression of AD. Epidemiologic research has reported that one-year delay in the onset of AD would reduce the projected global burden by 9 million [6]. Therefore, preclinical detection of neurodegeneration will be crucial for preventing dementia caused by AD and developing new treatments.

Neuroimaging is the most widely used method for early diagnosis of neurodegenerative pathology of AD. With the advent of different neuroimaging technologies, quantitative assessment of cerebral structures and metabolic states in specific regions has become possible. Both magnetic resonance imaging (MRI) and computed tomography (CT) can identify cortical and hippocampal atrophy. In addition, Aβ deposits can be quantified by amyloid positron emission tomography [7, 8]. Besides, the cerebrospinal fluid test has been thought to be able to reflect the pathology of AD through decreased amyloid and increased tau levels [9, 10]. The above methods, however, are invasive, expensive or time-consuming, which may not be suitable for early large-scale screening of AD. Thus, there is an urgent need for an early, noninvasive and cost-efficient tool to identify AD biomarkers, which could help detect AD pathology in mild cognitive impairment (MCI) stage, even in asymptomatic preclinical AD stage. Such strategy could help predict those at a high risk of developing dementia in a large-scale population.

As a window to the brain, the retina provides a unique opportunity to study the pathophysiology of many ophthalmic and neurodegenerative diseases. A growing body of evidence has indicated that both the brain and the retina are affected in AD and these pathologic changes are significantly correlated [11]. Some evidence has also shown that the retinal nerve fiber layer (RNFL) thinning, which reflects the loss of retinal ganglion cells (RGCs), is significantly associated with brain atrophy [12, 13]. The optical coherence tomography (OCT) has enabled the imaging and quantification of RGCs and their axons.

In addition to the neurological defects, evidence has shown that the vascular factors also play a crucial role in the occurrence and development of AD [14, 15]. With the advancement of technology, the retinal microvasculature can be visualized and quantified non-invasively using optical coherence tomography angiography (OCTA). Compared to the traditional color fundus photography, OCTA has advantages of visualizing retinal microvasculature at the micrometer level. OCT and OCTA imaging of the retina are potential screening tools for early diagnosis of AD and identification of the risk of disease progression. In this review, we summarize recent development on the applications of OCT and OCTA as retinal imaging tools to study the pathophysiology of AD at different stages. We also discuss the clinical implications of these findings and what we can do in future research.

## Why is the retina a window to the brain?

The retina originates from the neural tube as a part of the central nervous system during embryonic development [16]. The RNFL is mainly composed of axons, while the ganglion cell- inner plexiform layer (GC-IPL) mainly contains cell bodies and dendrites. In terms of the brain parenchyma, the white matter of the brain is mainly composed of axons, while the gray matter is mainly composed of cell bodies and dendrites of neurons. Research has demonstrated that thinning of the GC-IPL is significantly associated with the gray matter volume obtained from MRI scans, suggesting that the retinal ganglion cells are potential markers of cerebral neurodegeneration [17].

**Fig. 1:**
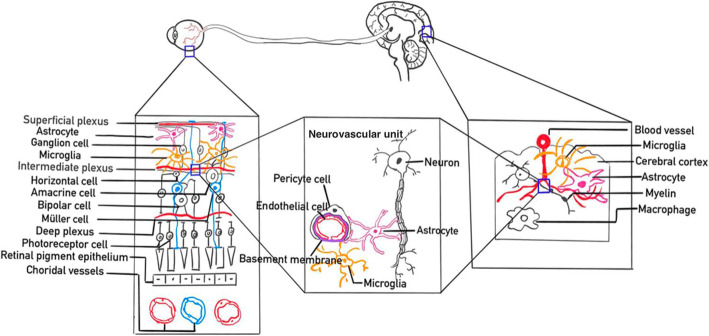
Schematic diagram of neurovascular units of the retina and brain

In terms of the retinal microvasculature around macula, the inner layer of retina is supplied by the ophthalmic artery through the central retinal artery. The ophthalmic artery originates from the internal carotid artery, similar to the intracranial artery. The external retina is supplied by choroid vessels. The blood supply of the optic disc is derived from the posterior ciliary artery circulation, with the retinal circulation supplying the surface nerve fiber layer [18–21]. The retinal arterioles and venules, with diameters from 100 μm to 300 μm, have similar anatomical features and physiological properties with cerebral small vessels, which provides a unique and accessible “window” to study the subclinical microvascular pathology of the brain (Fig. 1).

As one of the main pathological features of AD, Aβ is not only associated with blood vessels but also occurs in the retinal neural structure. Researchers have discovered that the retinal plaques are detectable earlier than those in the brain and accumulate with disease progression in animal models [22]. Koronyo Y and colleagues [11] detected curcumin-labeled Aβ plaques in patients *in vivo* and showed that the retinal Aβ burden was quantitatively correlated with the amyloid burden in the brain. With regard to other pathological states, patients with raised intracranial pressure often develop optic disc edema, and fundus findings including papilledema and hemorrhage are common in patients with spontaneous subarachnoid hemorrhage [23]. A large standard deviation in retinal arterial width is associated with intracranial arterial stenosis, which indicates that the retinal vessels may provide insights into the cerebrovascular network [24].

## Advances in imaging of retinal neural structure and microvasculature based on OCT and OCTA

OCT is an invasive imaging tool with advantages of high resolution, excellent repeatability, fast scanning speed, and high convenience, which can provide quantitative analyses of individual retinal layers, including the inner boundary membrane, RNFL, ganglion cell layer (GCL), inner plexiform layer (IPL), inner nuclear layer (INL), outer plexiform layer (OPL), outer nuclear layer (ONL), outer membrane, photoreceptor cell layer, and retinal pigment epithelium [25, 26]. The peripapillary RNFL (pRNFL), which converges to form the optic nerve, is composed of axons of ganglion cells and is thickest around the optic disc. The ganglion cells are mainly concentrated in the macular area. High-resolution spectral-domain OCT also allows measurement of the thickness of the pRNFL, the GCL, the GC-IPL, and the ganglion cell complex (GCC), which is composed of pRNFL and ganglion cell bodies and dendrites. The INL contains cell bodies of horizontal, bipolar and amacrine cells, and the ONL contains cell bodies of rods and cones. The two neuropils consisting of synaptic contacts divide the above nerve cell layers. The different degrees of light absorption and scattering in individual retinal layers serve as the basis for imaging. Compared with the conventional time-domain OCT devices, the spectral-domain OCT technology offers faster scanning speed and higher axial resolutions. Recently, swept-source OCT has been shown to be more accurate in quantitative segmented retinal layer analyses with increased speed and resolution. The current equipment and built-in software have enabled us to assess the thickness of each retinal layer in different quadrants of macula and optic disc within a minute.

Previous studies based on fundus photography have demonstrated that the quantitative retinal vascular parameters including fractal dimension (FD), central retinal artery equivalent, and central retinal vein equivalent, as well as the qualitative retinopathy, are associated with dementia [27, 28]. Nevertheless, findings from other studies are inconsistent, especially in the early stage of AD [29–32]. This discrepancy may be because that the relatively large blood vessels detected by the fundus camera were not sufficient to reflect the subtle pathology in the early stage of the disease.

As a milestone in the development of retinal imaging technology, the OCTA can detect the movement of red blood cells in the vascular lumen *via* changes in OCT signal measured by multiple scans in the same cross section. Combined with continuous en-face blood cell movement information, complete three-dimensional imaging of retinal and choroid vessels can be achieved. This is a novel imaging technique that can noninvasively and rapidly depict the microvasculature in different retinal layers with high resolution [33–36]. In addition to the retinal vessel density (VD), FD [37], which represents the retinal capillary complexity, can be obtained from OCTA to detect subtle microvascular changes in the early stage of retinopathies [38] and neurodegenerative diseases [39]. In the following, we summarize relevant studies of OCT/OCTA in AD, MCI and preclinical AD patients (Tables 1 and 2, respectively).

**Table 1:** Studies on OCT parameters in patients with AD, MCI and preclinical AD

Authors	Year of publication	Layer	Subjects	Alteration
Parisi et al. [40]	2001	pRNFL	AD	Thinning
Iseri et al. [41]	2006	pRNFL, mRNFL	AD	Thinning
Berisha et al. [42]	2007	pRNFL	AD	Thinner in the superior quadrant
Lu et al. [43]	2010	pRNFL	AD	Thinner in the superior and inferior quadrants
Moschos et al. [44]	2012	pRNFL	AD	Thinning
Kirbas et al. [45]	2013	pRNFL	AD	Thinner in the superior quadrant
Marziani E et al. [46]	2013	mRNFL mRNFL + GCL	AD	Thinning
Garcia-Martin et al. [47]	2016	pRNFL, mRNFL GCL, IPL, INL, OPL, ONL, RPE	AD	Thinner pRNFL, mRNFL, GCL, and IPL
Gharbiya et al. [48]	2014	pRNFL	AD	No difference
Larrosa et al. [49]	2014	pRNFL	AD	Thinning except the nasal quadrant.
Kromer et al. [50]	2014	pRNFL	AD	Thinner in the nasal superior sector
Polo V et al. [51]	2017	pRNFL	AD	Thinner in the superior and inferior quadrants.
Salobrar-Garcia et al. [52]	2015	pRNFL, mRNFL	AD	Thinner mRNFL
Eraslan et al. [53]	2015	pRNFL, GCC	AD	Thinning
La Morgia et al. [54]	2016	pRNFL	AD	Thinning
Cunha et al. [55]	2017	pRNFL	AD	Thinner in the temporal superior quadrants and globally
Liu et al. [56]	2015	pRNFL	AD	Mild and moderate AD had thinner pRNFL in the superior quadrant. Severe AD had thinner pRNFL in the superior and inferior quadrants.
Ferrari L et al. [57]	2017	pRNFL, pGC-IPL	AD MCI	No difference in mild AD. Moderate AD had thinner pRNFL and pGC-IPL. MCI had thinner pRNFL.
Paquet et al. [58]	2007	pRNFL	AD MCI	Thinning
Kesler et al. [59]	2011	pRNFL	AD MCI	AD had thinner pRNFL in the superior and inferior quadrants. MCI had thinner pRNFL only in the inferior quadrant.
Ascaso et al. [60]	2014	pRNFL	AD MCI	AD had thinner pRNFL in all sectors. MCI had thinner pRNFL in all sectors except the temporal quadrant.
Gao et al. [61]	2015	pRNFL	AD MCI	Thinning
Oktem et al. [62]	2015	pRNFL	AD MCI	Thinning
Cheung et al. [63]	2015	pRNFL, GC-IPL	AD MCI	AD had thinner pRNFL in the superior quadrant and thinner mGC-IPL in all sectors. MCI had thinner mGC-IPL in the majority of sectors.
Shao et al. [64]	2018	pRNFL, GC-IPL	AD MCI	Thinning
Almeida et al. [65]	2019	pRNFL, mRNFL	MCI	Thinner mRNFL, mGC-IPL and mGCC
Santos et al. [66]	2018	pRNFL, mRNFL, GCL, IPL, OPL, INL, ONL	Preclinical AD	Thinner mRNFL, ONL and IPL
López-Cuenca et al. [67]	2020	pRNFL, mRNFL, GCL, IPL, INL, OPL, ONL, RPE	Preclinical AD	Thinner mRNFL, IPL, INL and OPL
Snyder et al. [68]	2016	pRNFL, mRNFL, GCL, IPL, INL, OPL, ONL,	Preclinical AD	Thicker IPL
van de Kreeke et al. [69]	2019	pRNFL, mRNFL, GCL, IPL,	Preclinical AD	No difference
van de Kreeke et al. [70]	2020	pRNFL, mRNFL, GCL, IPL,	Preclinical AD	No difference in the rate of change of any retinal layers

*OCT* optical coherence tomography; *AD* Alzheimer’s disease; *MCI* mild cognitive impairment; *pRNFL* peripapillary retinal nerve fiber layer; *mRNFL* macular retinal nerve fiber layer; *GCL* ganglion cell layer; *IPL* inner plexiform layer; *GC-IPL* ganglion cell-inner plexiform layer; *pGC-IPL* peripapillary ganglion cell-inner plexiform layer; *mGCC* macular ganglion cell complex; *INL* inner nuclear layer; *OPL* outer plexiform layer; *ONL* outer nuclear layer; *RPE* retinal pigment epithelium

**Table 2:** Studies on OCTA parameters in patients with AD, MCI and preclinical AD

Authors	Year of publication	Parameters	Subjects	Alteration
Bulut M et al. [71]	2018	SRCP-VD, DRCP-VD, FAZ	AD	Lower VD, enlarged FAZ.
Lahme L et al. [72]	2018	SRCP-flow density, DRCP-flow density, Peripapillary-VD	AD	Lower SRCP-flow density
Jiang et al. [73]	2017	SRCP-VD, DRCP-VD	AD MCI	AD had lower VD of SRCP and DRCP. MCI had lower VD of DRCP in the superior nasal quadrant
Stephen P et al. [74]	2019	SRCP-VD, FAZ	AD MCI	AD had lower SRCP-VD. No difference in MCI
Zhang et al. [75]	2019	SRCP-VD, DRCP-VD, RPC	MCI or early AD	Lower SRCP-VD
Wu et al. [76]	2020	SRCP-VD, DRCP-VD, FAZ	AD MCI	Lower DRCP-VD
O’Bryhim et al. [77]	2018	SRCP-VD, FAZ	Preclinical AD	Lower VD, enlarged FAZ
van de Kreeke et al. [78]	2020	Macular-VD, Peripapillary-VD, FAZ	Preclinical AD	Higher VD, no difference in FAZ.

*OCT* optical coherence tomography; *AD* Alzheimer’s disease; *MCI* mild cognitive impairment; *SRCP-VD* superficial retinal capillary plexus-vessel density; *DRCP-VD* deep retinal capillary plexus-vessel density; *RPC* radial peripapillary capillary; *FAZ* foveal avascular zone

## Changes in retinal neural structure in AD, MCI, and preclinical AD

### Changes in RNFL and GCC in AD, MCI, and preclinical AD

The compositions of retinal RNFL and GC-IPL correspond to white and grey matter components of the brain, therefore, they are widely regarded as neural parameters reflecting the progression of AD. At advanced stage of AD, a recent meta-analysis also found a significant reduction in the overall mean pRNFL thickness as well as in the mean pRNFL thickness of all four quadrants [79]. In addition, some studies have reported significant pRNFL thinning in individual quadrants. In most studies, the superior [41–43, 45, 49, 51, 55, 61] and inferior [41, 43, 49, 51] quadrants showed significantly greater thinning in patients with AD compared with controls, whereas the temporal [49, 55] and nasal [41, 61] quadrants were found to be significantly thinner only in a small number of studies. This pattern of quadrantal change in the retina may indicate that the magnocellular ganglion cells located in the extramacular retina are vulnerable to AD pathology [54]. In addition, another explanation is that the concentration of axon bundles is highest in the superior and inferior quadrants [80]. The characteristic quadrantal loss in the RNFL may have better diagnostic efficacy in distinguishing different types of dementia. In a recent study, researchers divided AD patients into different subgroups (mild, moderate and severe AD) and found that the pRNFL degeneration paralleled dementia progression [56]. The pRNFL decreased significantly in the superior quadrant in mild and moderate AD patients, compared to the controls. In severe AD, the loss of pRNFL occurred not only in the superior quadrant but also in the inferior quadrant. Others have pointed out that the size of the RGC is 10 to 20 times the diameter of axons, which may lead to more significant changes in the thickness of GC-IPL and GCC compared to the pRNFL [63]. Several studies have also found changes in the GC-IPL and GCC in AD. Significant thinning of the average GCC has been associated with AD [53]. A recent meta-analysis also showed that the average thickness of GC-IPL was significantly decreased in AD and the thinning occurred in most sectors around the fovea, except the supratemporal sector [81]. In addition, a series of studies has shown that the cognitive scores are significantly correlated with macular parameters rather than the pRNFL thickness [41, 57, 65]. A large population-based study in Japan discovered that the presence of dementia was inversely associated with full macular thickness and GCC thickness but not with the pRNFL thickness [82]. Another population-based study in Germany also showed that the GCC volume was more strongly related to the global function than was the pRNFL thickness [83]. In line with the above results, some also inferred that the GCC thickness performed better than pRNFL thickness in detecting the disease status based on the area under the curve value [84]. The divisions of the retina seem to have better diagnostic performance due to the less influence from individual variation [85]. These results indicate that the macular parameters are more useful than peripapillary parameters.

Similarly, pRNFL, GC-IPL or GCC thinning has also been found in MCI patients [56–64, 79, 86]. Others even discovered thickening and thinning of regions adjacent to each other in MCI patients, indicating that the two layers undergo dynamic changes during progression from MCI to AD [87]. Interestingly, the mean thickness of GC-IPL and GCC in MCI patients is significantly reduced compared with that in controls, while changes in the pRNFL thickness are not significant [65]. The above findings suggest that the ganglion cells around macula, reflected by the thickness of GC-IPL or GCC, are sensitive in detecting neural structural changes in the initial stage of MCI.

In addition, a 27-month longitudinal study on preclinical AD showed significant changes in macular RNFL (mRNFL) rather than pRNFL over time [66]. The mRNFL thinning was significantly correlated with increased neocortical accumulation of Aβ and impaired performance in a task of audiovisual integration efficiency. Another study also suggested that the axonal loss in the mRNFL occurred earlier than pRNFL degeneration and this retinal parameter was expected to reflect the subtle disruption of white matter integrity in the preclinical AD stage [67]. However, several other studies did not report similar significant results [68, 69]. Current cross-sectional studies have reported no statistically significant differences in the pRNFL or GCL between preclinical AD and controls. A longitudinal follow-up study also showed no difference in the rate of change of the above layers [70]. Therefore, the diagnostic efficacy of retinal neural structural parameters and their relationship with the progression of AD should be further studied especially in the preclinical AD stage.

### Other retinal layer changes in AD, MCI, and preclinical AD

In additional to the neurodegenerative changes, neuroinflammation in retina and brain is also involved in AD progression [88]. Retinal glial cells including Muller cells, astrocytes and microglia cells are involved in immune and inflammatory responses and may lead to changes in the outer layer of the retina. The outer layer is relatively less studied and the results are inconsistent. Some studies in AD patients have found remarkable thickening of the ONL [64, 89], while others revealed no significant decrease of the outer retinal thickness including the ONL [90]. Furthermore, there is no significant difference in IPL and INL, albeit with OPL thinning [89]. However, inconsistent with the above results based on OCT devices, postmortem examination of retinas of AD patients showed thinning of pRNFL, GCL, IPL, INL, OPL and ONL [91]. The researchers further pointed out that the retinal structural damage may be attributed to the retrograde trans-synaptic degeneration, deposition of Aβ plaques, as well as the consequent neurotoxicity.

In MCI subjects, some studies have found OPL thinning and ONL thickening [89], while others revealed no significant difference in the ONL [90]. Only a few studies have focused on the outer retinal layers in preclinical AD and the results are controversial. A recent study found significant decreases in the IPL, INL and OPL in *ApoE* ɛ4 carriers with a family history of AD compared to the non-carriers without a family history [67]. Another study also showed a decrease in IPL and ONL volumes over a 27-month follow-up in older adults with multiple risk factors for AD, compared to the healthy control subjects [66]. Inconsistently, a cross-sectional study reported no significant differences in INL, ONL and OPL [68]. Besides, remarkable IPL thickening has been reported as well. As for IPL, a team showed no difference in cross-sectional observation or in the rate of change [69, 70]. These findings suggest that the individual retinal layers play different roles at different stages of AD, and the thickening and thinning of the outer layer, which contains a variety of cells, is not specific compared to the RNFL and GCC. Of course, the differences in diagnostic criteria, inclusion and exclusion criteria, and devices may lead to the heterogeneity of the results. Further large longitudinal studies are needed to confirm the effectiveness of individual retinal layers, especially in the preclinical AD stage.

### Retinal microvascular changes in AD, MCI and preclinical AD

Retinal microvascular OCTA findings are relatively less reported. In general, retinal microvascular damage can be observed at all stages of AD. A report on monozygotic twin pairs showed significantly decreased VD in the superficial retinal capillary plexus (SRCP) in twins with AD compared with cognitively normal twins, indicating that the changes of SRCP may serve as a possible biomarker for AD based on OCTA [92]. Several recent studies have also shown decreased SRCP or deep retinal capillary plexus (DRCP), as well as enlarged foveal avascular zone (FAZ) in AD subjects [71–74, 76]. The FAZ enlargement may be due to the dropout of vasculature within the fovea. Besides, a significantly reduced flow density in radial peripapillary capillaries (RPC) with larger vascular channels in the peripapillary region has also been found in AD patients [72]. Most studies found that the vascular parameters were significantly correlated with cognitive scores [71, 74–77]. However, some studies also showed no association between retinal vascular parameters and cognitive function [72, 73]. The inconsistency may result from ambiguous clinical staging of disease progression with a small sample size.

In addition, some studies have shown a tendency of microvascular density loss along the development of AD, which indicates that the retinal vascular impairment reflected as reduced microvascular density by OCTA can serve as a marker for monitoring disease progression [73]. Meanwhile, MCI individuals had significantly declined parafoveal SRCP VD and adjusted flow index, while no difference was found in the RPC [75]. Contrary to the above study, other studies have demonstrated no statistically significant difference in SRCP VD [73, 74, 76]. Interestingly, two studies found lower microvascular density in DRCP rather than in SRCP in MCI patients [73, 76]. This may be because that the smaller microvasculature of the DRCP is more susceptible to disease development than larger vessels of the SRCP, suggesting that the deep microvascular density around macula is more sensitive in detecting and monitoring progression of MCI at the early stage.

So far, there are only two relevant studies focusing on preclinical AD. Notably, one study revealed enlargement of the FAZ in individuals with preclinical AD with positive positron emission tomography scan biomarkers [77]. However, the other showed no statistical difference in FAZ size between preclinical AD and healthy individuals. Even more, they found increased vessel density in preclinical AD subjects, which may be caused by the increased blood flow secondary to hypoxia [78].

In sum, research on the role of microvascular pathology as reflected by OCTA in early diagnosis of AD is still in the preliminary stage. The relationship between vascular change and neurodegenerative changes needs to be further investigated in more studies with longer follow-up durations.

## Conclusion

In conclusion, accumulating evidence has shown that the retina could provide valuable insights into the early diagnosis of AD. Retinal neuronal structural and microvascular imaging by OCT/OCTA is potentially useful for large-scale population screening or monitoring responses to therapies in AD patients. Before full application in clinical practice, further research is needed to validate whether the findings on retinal neuronal loss and microvascular damage in MCI or even in preclinical AD patients are related with the progression of cognitive impairment and brain neuronal/vascular loss in individual AD patients, based on longitudinal data of multi-center studies. Histopathological studies are also needed to understand the pathophysiological mechanisms underlying the retinal neuronal and microvascular alterations, which will facilitate the clinical applications of retinal OCT/OCTA imaging in large-scale population screening and monitoring and the development of new therapies.

## Data Availability

Not applicable.

## Abbreviations

AD: Alzheimer’s disease
MCI: Mild cognitive impairment
OCT: Optical coherence tomography
OCTA: Optical coherence tomography angiography
MRI: Magnetic resonance imaging
CT: Computed tomography
RNFL: Retinal nerve fiber layer
GCL: Ganglion cell layer
IPL: Inner plexiform layer
INL: Inner nuclear layer
OPL: Outer plexiform layer
ONL: Outer nuclear layer
GC-IPL: Ganglion cell-inner plexiform layer
GCC: Ganglion cell complex
pRNFL: Peripapillary retinal nerve fiber layer
mRNFL: Macular retinal nerve fiber layer
VD: Vessel density
FD: Fractal dimension
FAZ: Foveal avascular zone
VLD: Vessel length density
RPC: Radial peripapillary capillary
SRCP: Superficial retinal capillary plexus
DRCP: Deep retinal capillary plexus
Aβ: Amyloid-beta
